# Epidemiology of emergency ambulance service calls related to mental health problems and self harm: a national record linkage study

**DOI:** 10.1186/s13049-019-0611-9

**Published:** 2019-03-20

**Authors:** Edward A. S. Duncan, Catherine Best, Nadine Dougall, Silje Skar, Josie Evans, Alasdair R. Corfield, David Fitzpatrick, Isabella Goldie, Margaret Maxwell, Helen Snooks, Cameron Stark, Chris White, Wojtek Wojcik

**Affiliations:** 1 Faculty of Health Sciences and Sport, University of Stirling, Stirling, UK; 2 School of Health and Social Care, Edinburgh Napier University, Edinburgh, UK; 3 Department of Emergency Medicine, NHS Greater Glasgow and Clyde, Glasgow, UK; 4 Mental Health Foundation Scotland, Glasgow, UK; 5 Medical School, Swansea University, Swansea, UK; 6 Department of Public Health, NHS Highlands, Inverness, UK; 7 Department of Liaison Psychiatry, NHS Lothian, Edinburgh, UK

**Keywords:** Mental health, Emergency department, Pre-hospital

## Abstract

**Background:**

People experiencing a mental health crisis receive variable and poorer quality care than those experiencing a physical health crisis. Little is known about the epidemiology, subsequent care pathways of mental health and self-harm emergencies attended by ambulance services, and subsequent all-cause mortality, including deaths by suicide. This is the first national epidemiological analysis of the processes and outcomes of people attended by an ambulance due to a mental health or self-harm emergency. The study aimed to describe patient characteristics, volume, case-mix, outcomes and care pathways following ambulance attendance in this patient population.

**Methods:**

A linked data study of Scottish ambulance service, emergency department, acute inpatient and death records for adults aged ≥16 for one full year following index ambulance attendance in 2011.

**Results:**

The ambulance service attended 6802 mental health or self harm coded patients on 9014 occasions. This represents 11% of all calls attended that year. Various pathways resulted from these attendances. Most frequent were those that resulted in transportation to and discharge from the emergency department (*n* = 4566/9014; 51%). Some patients were left at home (*n* = 1003/9014 attendances, 11%). Others were admitted to hospital (*n* = 2043/9014, 23%). Within 12 months of initial attendance, 279 (4%) patients had died, 97 of these were recorded as suicide.

**Conclusions:**

This unique study finds that ambulance service and emergency departments are missing opportunities to provide better care to this population and in potentially avoidable mortality, morbidity and service burden. Developing and testing interventions for this patient group in pre-hospital and emergency department settings could lead to reductions in suicide, patient distress, and service usage.

## Introduction

9,700,000 emergency calls were made to ambulance services in England and Scotland in 2014–2015. Patients who are experiencing a mental health emergency or who have self-harmed account for a substantial proportion (10%) of these calls [1, 2]. The care pathways and outcomes of the people attended by the ambulance service for a mental health crisis is currently unknown. It is plausible that people who are attend by the ambulance service for a mental health related emergency are at increased risk of suicide but this has not been previously investigated. Suicide is a pressing international public health issue with an estimated 804,000 people dying worldwide in 2012, corresponding to a standardised mortality rate (SMR) of 11.4 per 100,000. The SMR rate for the UK corresponds to 10.1 per 100,000 [3]. In the UK, as in many countries around the world, the leading cause of death in young people is suicide, and the Scottish Government declared suicide to be a significant public health concern, and following investment in suicide prevention, rates between 2002 and 2006 and 2013–2017 fell by 20% [4], equating to a SMR of 13.5 per 100,000 [5]. However, Scotland has disproportionately higher rates of suicide than its neighbouring country England, and for young men even more notably so [6], with corresponding significantly increased suicidal behaviour and suicide attempts observed in women [7]. Therefore the Scottish Government along with other national governments and the World Health Organisation [8] has renewed its commitment to suicide prevention and enshrined several strategic aims, including one where ‘people at risk of suicide feel able to ask for help, and have access to skilled staff and well-coordinated support’ [4]. Ensuring effective processes and positive outcomes for people experiencing a mental health crisis is essential. However quality of services and health outcomes for people who experience mental health crisis are highly variable and compare poorly to those received by people requiring a physical health emergency response [9]. The UK national mental health charity “Mind” has reported emergency care as inappropriate for people experiencing a mental health crisis, and staff as being unsympathetic to people’s situations [10].

This study investigates the epidemiology of mental health emergencies, including self-harm using Scottish Ambulance Service patient records. The Scottish health care system is a publicly funded National Health Service. The Scottish Ambulance Service is its national frontline emergency service. It provides an emergency ambulance service for the whole of Scotland, responding to over 600,000 emergency incidents per annum (2011 population 5.3 million). The study uses data from all calls to the Scottish Ambulance Service in 2011 to identify what proportion are for mental health related emergencies and then, to describe:- a) the characteristics of the population; b) their current care pathways; c) their clinical outcomes.

The aim of this study is to summarise information for people in contact with the ambulance service with mental health emergencies, so that opportunities are identified to develop interventions to decrease deaths by suicide, reduce distress, and avoid unnecessary emergency ambulance and emergency department contacts.

## Method

### Study design and setting

We conducted a retrospective cohort study of all patients attended by the Scottish Ambulance Service in 2011 with diagnostic codes relating to mental health emergencies or self-harm. We included all patients resident in Scotland and selected adults aged 16 or more years of age on first contact (as this is the age of legal capacity). The cohort was followed via linked records for at least one year, to the end of 2012. As the data have national coverage, there is no inherent selection bias.

### Data sources and pathway definitions

Data were extracted from the NHS Information Systems Division (ISD) Unscheduled Care Data Mart (UCDM) [11]. This provides person level linked-data using established probabilistic and deterministic matching techniques from the Scottish Ambulance Service, emergency departments (A&E2), general and psychiatric inpatient and day case hospital admission episodes (Scottish Morbidity Records SMR01 & SMR04) and death records, based on unique identifiers including NHS number, name and date of birth. The years 2011–2012 were selected as this was the most recent period in which the ambulance service perceived there to be reasonable data quality. Data were transferred and accessed through a secure connection to a data safe haven hosted by the Farr Institute and facilitated by ISD.

The data are organised in continuous care pathways. This includes all service use where contact with one service happens within 24 h of the previous service. Each service contact is denoted by a letter: Scottish Ambulance Service is “S”; Emergency Department, “E”; acute hospital services, “A”; and mental health services “M”. Therefore a pathway that involved attendance by an ambulance, then transport to the emergency department, then discharged and then return to the emergency department within 24 h would be coded “SEE”. Pathways included in the data set were truncated at 11 characters. One pathway could have more than one mental health emergency call. In these cases the pathways were duplicated in the data e.g. SEESE appeared as two rows in the data with the first connected to information on the first call and the second to the second.

### Derivation of variables to identify psychiatry emergencies and episodes of self-harm

The Scottish Ambulance Service uses a triage system called Advanced Medical Priority Despatch System (AMPDS) to provide symptom based intervention, record patients’ most important condition, prioritise calls and allocate appropriate resources. An ambulance control centre call handler assigns an initial AMPDS code and an attending ambulance clinician confirms or amends this code following face to face assessment, thus producing ‘final AMPDS’ codes, which this study used. AMPDS codes are systematically checked by ambulance clinicians for all calls and updated as required. For this study we accessed only the final AMPDS codes, and therefore it was not known what proportion of calls switched from initial to final AMPDS codes. Furthermore, if mental health or self harm was identified in the narrative data, but not in the codes used these cases were not identified. Relevant patient attendances were identified using the list of Scottish Ambulance Service AMPDS code descriptions used by ambulance clinicians on attendance with the patient. A Scottish Ambulance Service lead with extensive knowledge and experience of using AMPDS in practice, met with members of the study team (EAD, DF, ND & SS) to select relevant codes. Only codes identified as directly relating to ambulance service psychiatric emergencies or self-harm were included in the data extraction. Codes were excluded if they could be used to describe a patient emergency or episode of self-harm (e.g. haemorrhage), but which could also relate to irrelevant cases. Following discussion and analysis of the complete data set the following codes were included:- 09E03 (Hanging); 17D02J (Falls, long fall (= > 6 ft./2 m) – Jumper); 17D03J (falls, unconscious or not alert – Jumper); 23 (intentional poisoning); 25A01 (psychiatric, non-suicidal without 1st party verification (alert & awake); 25B01 (psychiatric, serious haemorrhage); 25B02 (psychiatric, minor haemorrhage); 25B03 (psychiatric, threatening suicide); 25B04 (psychiatric, jumper (threatening)); 25D01 (psychiatric, not alert). These codes therefore aimed to capture people clearly presenting with psychiatric emergencies, suicidal or self-harming behaviour, and were intended to maximise specificity to detect relevant records. Linked data relating to the selected AMPDS codes were extracted and placed in the safe haven for analysis.

The data received were arranged by mental health call. That is, there was one row in the spreadsheet for each care pathway containing a mental health call. Each person had a unique identifier and each person had more than one row in the table if they made more than one call. For each mental health call an individual’s care pathway could have multiple visits to ED and multiple admissions to hospital and we received data regarding the dates and times of all these visits. To define the index sample we identified all mental health calls made in 2011. The care pathways were sorted by frequency. The times between the index call and each subsequent point on the care pathway was calculated (i.e. time from call to arrival at ED, time in ED etc.)

To determine the number of repeat calls that were made within one year of the index call we reverted to the full data set (not just calls in 2011). The data were converted into person-period (wide) format with one row per person. Then the people who did not make their first call in 2011 were dropped. The times between all calls by the same individual were calculated to determine how many were made within 1 year of the index call.

In common with other routinely collected data studies [12], the data required to be cleaned to remove spaces and reconfigure the data in a format suitable for analysis. Algorithms were written using Stata to prepare the data for analysis. This Stata software code has been submitted to the GitHub, an Open Acess Data Repository (https://github.com/CathBest/PEPP-study). This is an online archive of methods, models and codes relevant to processing administrative data. Code is freely available within the archive for other researchers to use.

### Pathway analysis

We began the pathway analysis after the first included ambulance service attendance of 2011 to determine the most common care pathway that occurred after someone had called ambulance service. We identified the four most common pathways and combined longer and more complex pathways into an ‘other’ category.

### Statistical analysis

Frequencies for categorical variables were reported as counts and percentages of all people or calls. For continuous variables the degree of deviation from the standard normal distribution was visually assessed. In all cases distributions were found to be non-normal. Therefore continuous variables were summarised by median and interquartile range. Bivariate relationships were analysed using chi squared tests for categorical variables and non-parametric Mann Whitney U tests for continuous variables.

## Results

The results have been reported in keeping with the RECORD checklist for studies conducted using observational routinely collected data [13] and the STROBE reporting guidelines for observational studies [14].

### Cohort characteristics

There were 9014 (out of around 500,000) calls from 6802 people for mental health emergencies in Scotland during 2011. This accounted for 11% of all-cause calls to the ambulance service. Slightly more males (*n* = 4708, 52%) than females (*n* = 4306, 48%) were attended.

Table 1 presents the distribution of included calls by AMPDS description; almost half of the attendances were designated as ‘alert & awake’ (4315, 48%). Of all 9014 calls: 1816 (20.15%) were additionally tagged as alcohol-related; and 94 calls (1.04%) were tagged as drug-related.

**Table 1:** Reasons for emergency call

	Overall sample		Direct ambulance to Emergency Department transfers	
Final AMPDS Description	*N*	%	*N*	%
Psychiatric, non-suicidal without 1st party verification (alert & awake)	4315	48	3338	45
Psychiatric, minor haemorrhage	2615	29	2307	31
Psychiatric, serious haemorrhage	1117	12	955	13
Intentional poisoning	697	8	639	9
Psychiatric, threatening suicide	129	1	120	2
Hanging	43	< 1	15	< 1
Psychiatric, jumper (threatening)	79	< 1	59	< 1
Falls, long fall (= > 6 ft./2 m) – Jumper	< 15	< 1	< 15	< 1
Falls, unconscious or not alert - Jumper	< 15	< 1	0	0
Psychiatric, not alert	< 15	< 1	< 15	< 1
TOTAL	9014	100	7450	100

### Patient pathways

A total of 6802 people made an emergency call for a mental health or self harm problem and were attended by ambulance clinicians, in Scotland, in 2011 (Table 2). These people were followed up for 1 year each after their index attendance. Just over half of the people (52%, *n* = 3564) had only the one index attendance. However, 3238 (47.6%) people were attended more than once, though not all calls were necessarily directly mental health related. Almost one fifth had a second attendance within 12 months (19%, *n* = 1294), leaving almost one third of people with three of more attendances (29%, *n* = 1944). One hundred people (1.5%) were attended by the ambulance service more than 16 times.

**Table 2:** Number of additional attendances to people who called for an ambulance for a mental health emergency within one year of the first call

Number of repeat attendances in 12 months	No. of people who called/ were attended to	Percent
0 (index call only)	3564	52.4
1	1294	19.02
2	646	9.5
3	366	5.38
4	218	3.2
5–9	459	6.75
10–14	155	2.28
15 to 101	100	1.47
Total	6802	100

More than half of the people attended by the ambulance service (*n* = 4169/6802, 61.3%) were either discharged from an Emergency Department with no known follow -up (*n* = 3369/6802, 49.6%) or left at home, most likely because they refused to be transported to hospital, (*n* = 800/6802, 11.8%) (Table 3). (IQR: 87.4–198.8). The median length of stay for patients who were taken to the Emergency Department was 140 mins (IQR 87 to 199 mins). ﻿Almost a fifth of care pathways that ended in transfer to Emergency Departments resulted in self-discharge (n = 607/3615, 17%)﻿. People who self-discharged from the Emergency Department had a Median length of stay that was 50 min shorter than those who did not (100.5 mins (IQR 52.4–150.7) vs 150.7 mins (IQR 98.3–209.7); two-sample Wilcoxon rank-sum test z = 14.64 *p* < 0.001. People who self-discharged from the Emergency Department before completion of treatment were 25% more likely to make another call to the ambulances service for a mental health emergency within the same year (Pearson chi square = 5.24, *p* = 0.02 RR = 1.25) than people who completed treatment. Patients were 49% more likely to self-discharge if they were intoxicated with alcohol (person chi squared = 35.4, p < 0.001 RR = 1.49). People admitted to an acute ward setting stayed for a median of one day, while people admitted to a psychiatric service stayed for a median of eight days.

**Table 3:** Pathway by first call for people who were attended by ambulance clinicians, along with details of repeat attendances for those individuals, and mortality during the following year after first attendance

Pathway of 1st call	Pathway Frequency *N* (%)	Lowest SAS priority by final AMPDS code *N* (% Pathway Frequency)	Additional calls to ambulance service *N* (% Pathway Frequency)	Deaths within 1 day of call *N* (% by pathway)	Deaths > 1 day and < 1 year *N* (%people by pathway)
S	800 (11.8%)	498 (62.3%)	321 (40.1%)	29 (3.6%)	22 (2.8%)
SE	3369 (49.6%)	1579 (46.8%)	1630 (48.4%)	< 15	80 (2.4%)
SEA	995 (14.6%)	402 (40.4%)	455 (45.7%)	< 15	50 (5.3%)
SEM or SM	656 (9.7%)	354 (54.0%)	251 (38.3%)	0	27 (4.1%)
Other	982 (14.4%)	470 (48.1%)	581 (59.2%)	< 15	61 (6.2%)
Total	6802 (100%)	3303 (48.6%)	3238 (47.6%)	39 (0.6%)	240 (3.5%)

*S* Ambulance, *E* ED, A Acute admission, *M* Psychiatric hospital admission

### Mortality outcomes at 1 year

279 (4.1%) of the 6802 people attended by an ambulance for a mental health emergency in Scotland in 2011 died within one year of first attendance. Just over one third of these 279 deaths were confirmed suicide (*n* = 97; 34.8%). Of note, recording of suicides differs between Scotland and England and Wales. Whilst this may theoretically lead to slightly higher reporting of suicide in Scotland (as opposed to open or narrative verdicts in England), overall data have been shown to be comparable, and there is a higher rate of suicides in Scotland [5]. The remainder were attributed to ‘accidents’,  ‘mental and behavioural disorders’ (e.g. dementia, alcohol dependence and drug dependence) (*n* = 64; 23%), and long term conditions (e.g. malignant neoplasm; heart disease; diseases of the respiratory system) with co-morbid psychological distress that were not directly mental health related (*n* = 118; 42%).

In order to examine cases where there may be the potential for upstream intervention to reduce mortality, deaths more than one day after attendance by the ambulance service were examined, and found to number 240 (3.9%) of 6802 people after the index emergency mental health attendance. The highest rate of death by pathway was in those left at home by the ambulance (*n* = 51;6.4%), while the pathway with the highest number of deaths occurred in those transported and discharged from the Emergency Department (*n* = 80; 1.3% of 6802 people). Therefore, almost half of all deaths > 1 day and < 1 year of call (*n* = 102; 43%) were either not taken to hospital by the ambulance service (S) or were discharged from ED (SE). One quarter died by suicide (*n* = 59; 25%) as confirmed by the corresponding death certificate for each person, of whom almost half (*n* = 27; 46%) had previously been left at home by the ambulance service or discharged from an Emergency Department with no follow-up recorded within the hospital notes.

## Discussion

### Key findings

This is the first UK study to report the epidemiology of emergency ambulance attendances for mental health emergencies including self-harm, including linked record outcomes. Most people attended by the ambulance service were either left at home or transported to and discharged from an Emergency Department. Though just over half of all people were only attended to once, repeat calls within 12 months were relatively common. People who were transported to ED but then self-discharged before completion of treatment were statistically more likely to make another emergency call to ambulances service within the same year. People were more likely to self-discharge themselves from the Emergency Department if they were intoxicated with alcohol.

Within 12 months of their first emergency call, 279 people (4.1%) had died, 97 (35%) recorded as suicide. People who were still alive after one day of index attendance numbered 240, of whom 59 were confirmed suicide. Given that in Scotland 772 people died by suicide in the year 2011, this suggests the ambulance clinicians were in contact with about 8% of these individuals.

### Comparison with other research

The proportion of attendances that were deemed to be psychiatric emergency or self-harm related was 11% of the final AMPDS codes of all-cause calls to the Scottish Ambulance Service. This compares with research reported for Victoria, Australia by Roggenkamp et al. [15], which estimated 9.5% of emergency attendances were mental health-related. A consideration in comparing these papers is that Scotland has a national health service, including the national ambulance service which is free to access at the point of contact, therefore incentives to gather additional data related to dedicated service utilisation, or secondary diagnoses on which people could be billed may not be present, and may explain some variation. With respect to gender we found just under half of calls (48%) were for women, this compares with Roggenkamp et al. [15] who found that the majority of attended calls were for women (57%), further suggesting different geographical and healthcare contexts are also at play.

### Limitations

In common with other record linkage studies [16], this study experienced problems with data quality and accuracy. Missing data was most evident from the A&E2 and SMR1 data sets and this limited our ability to report demographic information on the sample e.g. ethnic group and area-level deprivation indices based on postcode. However data were complete for the outcomes reported in this paper. We used a data set from 2011, which was the most recent reliable year in which data was available at the time of the study. The passage of time and the increased focus of tackling stigma related to psychiatric emergencies and self-harm in Scotland may have altered current practice. We also found that data quality was variable across the differing datasets. Data completion was poorest in the Emergency Department (A&E2 data set) where missing triage data (33.9%) meant that it was not possible to calculate meaningful comparisons between ambulance and emergency department. The original figures provided to us by the Scottish Ambulance Service estimated an annual frequency of 30,000 mental health emergency related calls. Our study identified 9014 calls that we could be confident related to a mental health or self-harm emergency. This discrepancy occurred as a result of the codes that were included as relevant. Ambulance clinicians code calls by the predominant presenting condition. Therefore instances of mental health emergency or self-harm may not be recorded where these took second place to more urgent symptoms. In selecting the strictest criteria of the index event codes we recognise that we have a highly specified cohort at the expense of increased sensitivity to detect many relevant vulnerable people. Our estimates of outcome and service burden are therefore at the lower end of the possible range. Alcohol intoxication and substance misuse are not currently recorded as an AMPDS code. Instead these are separate optional field items that can be coded on the ambulance service electronic patient record as appropriate. Anecdotal feedback suggests that these items are not immediately apparent and their optional completion means that they are frequently not completed. It is therefore likely that the reported frequencies of alcohol intoxication and drug misuse are under reported, though we are unable to state what the missing proportion of cases are.

### Implications for practice

Despite the study limitations the results have important implications for practice. Suicide is a leading cause of death in young people and a globally significant public health concern. Scotland is no exception with 746 deaths reported in 2013 equating to a standardised mortality rate of 13.5 per 100,000 [12]. Delivering effective suicide prevention strategies is challenging, as detecting people at risk of suicide is difficult. Our study shows that ambulance clinicians are well placed to identify people at risk of suicide. Just over 1% of people in the study cohort (97/6802) died by suicide within 12 months of their initial presentation to the ambulance service. However, this represents approximately 13% of all people who died by suicide in Scotland within the same time period [12]. The ambulance service therefore have a unique opportunity to deliver a range of potential suicide prevention strategies through developing alternative care pathways to specialist mental health services, delivering suicide prevention interventions, and registering risk of suicide on admission to the Emergency Department.

The high levels of patient repeat mental health emergency calls, and frequency of self-discharges from Emergency Departments further emphasises the importance of developing evidence-based interventions to improve outcomes and reduce service burden. These findings may be explained by qualitative research that suggests that people who experience a psychiatric emergency or have self-harmed often feel that their needs are not met within an Emergency Department [10, 17]. The risk of people with a self-harm history self-discharging from Emergency Departments has, however, been known for over a decade [18]. Action should not be further delayed. Understanding both patients’ experience of Emergency Departments and practitioners’ perceptions of their competencies in dealing with this patient population would support intervention development, enable improvements in patient experience, and reduce rates of self-discharge and suicide.

Ambulance services are rapidly evolving to improve patient outcomes and reduce Emergency Department burden [16]. This places additional requirements and pressures on ambulance clinicians to make complex clinical judgements and decisions that were previously not required. Understanding effective and efficient methods to undertake service redesign and develop effective pre-hospital interventions are urgently required. In response to this need, the Scottish Government is currently piloting a Distress Brief Intervention Programme (www.dbi.scot) that has been developed as a time limited and supportive problem solving intervention for this patient population. The aim is that this programme will improve patient outcomes and reduce demand on the ambulance service and emergency departments [19].

The importance of pre-hospital care settings in the development of suicide prevention strategies has been largely overlooked in Scottish suicide prevention policy to date [16]. Evidence-based suicide prevention strategies should be developed or adapted for pre-hospital emergency care settings. Ambulance services have a unique opportunity to target an identifiably vulnerable cohort and deliver a range of potential prevention strategies that could result in a meaningful decrease in suicides in Scotland. The development of effective interventions with this patient population would contribute to the achievement of NHS England [20] and Scottish Government [21] policy objectives of reducing the number of mental health presentations to Emergency Departments.

Improvements to the processes and outcomes of people who call the Scottish Ambulance Service due to a mental health emergency are required. Half (62%) of the study cohort were either not taken to Emergency Department or were discharged from there with no recorded follow-up. Seventeen percent of calls that ended in an Emergency Department self-discharged prior to completion of their assessment or treatment. These people were more likely to make repeat calls and were potentially at greater risk of death by suicide. Focusing attention on this vulnerable group is likely to lead to improved patient outcomes and decreased service burden.

### Future research

Linked data analysis using detailed data-sets such as the ISD unscheduled care data-mart can provide meaningful sources of clinical and service information that can be used to support intervention development and service reorganisation. The method used in this study could be replicated with other clinical conditions. The data from this study were presented at a key stakeholder workshop. The findings of this workshop will be published elsewhere. Data, however, only tell part of the story. Further study is required to understand patients’ experience of Emergency Departments and practitioners’ perceptions of their competencies in dealing with this patient population. Such knowledge would support future intervention development that should then be tested through pragmatic clinical trials.

## Conclusion

In conclusion, the Scottish Ambulance Service dealt with over 9000 cases relating to a mental health emergency, including self-harm, in 2011. Both the ambulance service and Emergency Departments are currently missing opportunities to provide better care to this vulnerable population. This results in potentially avoidable mortality, increased levels of patient morbidity, and service burden. Developing and testing interventions and alternative care pathways for this patient group in pre-hospital and Emergency Department settings is urgently required. These have the potential to reduce suicide, patient distress, and service usage.
